# Addressing the Symptom Management Gap in Patients With Cancer and Heart Failure Using the Interactive Voice Response System: A Pilot Study

**Published:** 2018-03-01

**Authors:** Anecita P. Fadol, Tito R. Mendoza, Daniel J. Lenihan, Donna L. Berry

**Affiliations:** 1 Southwest Virginia Cancer Center, Norton, Virginia;; 2 Department of Symptom Research, The University of Texas MD Anderson Cancer Center, Houston, Texas;; 3 Department of Cardiovascular Medicine, Vanderbilt University Medical Center, Nashville, Tennessee;; 4 Phyllis F. Cantor Center for Research in Nursing & Patient Care Services, Dana-Farber Cancer Institute, Boston, Massachusetts

## Abstract

Patients with cancer and concurrent heart failure (HF) have severe symptoms that may adversely affect patients’ quality of life in addition to limiting effective anticancer therapy. A system of frequent monitoring could alert clinicians, providing the opportunity for timely intervention before patients become severely ill and require hospitalization. The purpose of this pilot study was to evaluate if the MD Anderson Symptom Inventory–Heart Failure (MDASI-HF) instrument preprogrammed via the interactive voice response system (IVRS) can be used to collect symptom data that will generate symptom alerts to providers based on preset severity levels. Twenty-six patients were enrolled in the study. Symptoms were monitored using the MDASI-HF delivered via IVRS on a weekly basis for 3 months. When a participant’s reported symptom(s) reached critical predetermined threshold levels, an alert prompted the nurse to triage the patient’s response and initiate interventions per protocol. Descriptive statistics were used to describe the ratings of symptom severity and symptom interference with daily function. Demographic and disease characteristics were summarized using means, standard deviations, ranges, count, and proportions. Paired t-tests were used to examine symptom reduction from baseline to the end of 3 months. Fourteen (54%) participants completed the study with average IVRS usage rates of 84% at 1 month and 82% at 3 months. Over the course of the IVRS monitoring, 152 IVRS calls were completed and 107 critical threshold alerts were generated, prompting physician notification, medication titration, and non-routine clinic visits. Most of these alerts were managed by telephone, particularly those related to diuretic titration, and prevented hospital readmission. Symptom monitoring via the IVRS can potentially bridge the gap in symptom management to improve clinical outcomes in patients with cancer and HF. The IVRS can be of benefit in the symptom management of patients, especially those constrained by geographic location. This can potentially improve the quality of care, patient satisfaction, and quality of life of these patients.

Symptom management in patients with cancer and concurrent heart failure (HF) presents a major challenge to patients, families, and health-care providers throughout the entire trajectory of the disease process (Fadol et al., 2008). In the general population, HF diagnosis is borne by individuals aged ≥ 65 years (Bleumink et al., 2004). Since cancer incidence also increases exponentially with advancing age (Berger et al., 2006), it is increasingly likely that a patient may have a coexistent cancer and HF. Cancer patients and cancer survivors are at a significant risk for the development of HF as a result of treatment-related cardiotoxicity (Yeh & Bickford, 2009) secondary to chemotherapy (Choueiri et al., 2011; Feola et al., 2011), radiation therapy (Schellong et al., 2010), and biotherapy (Pina, Souza, Duarte, Alvarez, & Miranda, 2012).

Heart failure resulting from chemotherapy-induced cardiotoxicity may occur acutely during chemotherapy administration or can manifest within a year or even decades after the initiation of therapy (Lipshultz et al., 2005; Pai & Nahata, 2000). Heart failure and cancer are progressive and complex disease conditions, per se. However, when both conditions occur concurrently in the same individual, the symptoms can be debilitating and may adversely affect the patient’s quality of life (Mulrooney et al., 2009), in addition to limiting effective anticancer therapy. Moreover, there is an overlapping of symptoms resulting from the cancer itself, cancer treatment, and HF, which makes symptom differentiation very difficult. For example, fatigue, which is considered a cardinal symptom of HF, can also result from cancer, metastatic disease, and cancer treatment toxicity. Exacerbation of HF is a major cause of hospitalization among Medicare recipients (Go et al., 2014). Approximately 25% of all patients hospitalized for HF are readmitted to the hospital within 30 days after discharge because of recurrence of symptoms (Keenan et al., 2008; Jencks, Williams, & Coleman, 2009). Monitoring of symptoms, regardless of etiology, is paramount in patients with both cancer and HF to assist clinicians in the management of these complex patients.

The recent Institute of Medicine (IOM) report, "Delivering High-Quality Cancer Care: Charting a New Course for a System in Crisis," identified the need to address the complex care needs of persons who have multiple coexisting diseases, increased side effects from treatment, and greater need for social support (IOM, 2013). Patients experiencing concomitant HF and cancer are illustrative of these complex needs. According to this IOM report, successfully addressing the complex needs of cancer patients requires care delivery that engages patients, uses learning health-care information technology, translates evidence into clinical practice, is subject to quality measurement and performance improvement, and is accessible and affordable for patients.

Engaging patients often involves frequent communication between patients and health-care professionals for the optimal management of symptoms. A frequent monitoring system could alert clinicians to early HF decompensation, providing the opportunity for intervention before patients become severely ill and require hospitalization. Increasing the patient’s access to health-care providers through not only face-to-face visits but also distance monitoring is a care strategy that may bridge the "quality chasm" described by the IOM (Annema, Luttik, & Jaarsma, 2009). 

## TELEMONITORING

There are a number of communication devices available using specialized computer technologies to collect patient information remotely between ambulatory care visits. Telemonitoring represents one strategy for the remote surveillance of patients with HF and cancer. Telemonitoring can be accomplished by means of a telephone-based interactive voice response system (IVRS) that collects information daily about symptoms reported by the patient. In a randomized controlled trial in lung cancer patients who had cancer-related thoracotomy, Cleeland and colleagues (2011) found that using IVRS for symptom monitoring at home after a hospital discharge resulted in a greater reduction in symptoms threshold events than it did for controls (19% vs. 8%, respectively). Similarly, a systematic review and meta-analysis of randomized controlled trials for HF comparing telemonitoring or structured telephone interview to usual care in 8,323 patients with chronic HF showed that telemonitoring reduced all-cause mortality, reduced HF-related hospitalizations, improved quality of life, and reduced health-care costs (Inglis, Clark, McAlister, Stewart, & Cleland, 2011). Moreover, structured telephone support for patients with chronic HF reduced the rate of death from any cause by 44% and the rate of HF-related hospitalizations by 21% (Inglis et al., 2010). However, a recent multicenter, randomized controlled trial involving 1,653 patients with HF comparing telemonitoring vs. usual care found no reduction in the risk of readmission or death from any cause (Chaudhry et al., 2010). Patients with cancer and concurrent HF were not studied in this trial, suggesting that telemonitoring in patients with complex comorbid conditions needs further study (Chaudhry et al., 2010).

The purpose of this pilot study was to describe the use of the MD Anderson Symptom Inventory–Heart Failure (MDASI-HF) instrument, preprogrammed via the IVRS to collect symptom data and generate symptom alerts to providers based on preset severity levels.

## METHODS

**Participants and Setting**

This pilot study was conducted at a National Cancer Institute–designated comprehensive cancer center in the southwest United States after institutional approval was obtained. Patients were able to participate if they had dual diagnoses of cancer and concurrent HF (newly diagnosed or with previous diagnosis); were 18 years and older; provided informed consent; were able to read, write, and understand English; had a working telephone number; and lived within the 100-mile radius of the cancer canter while enrolled in the study for the 3-month duration. A total of 117 patients were screened, with 44 (38%) meeting inclusion criteria. Of those, 26 (59%) agreed to participate. The remaining 18 (41%) patients refused to participate, primarily due to problems with transportation to the hospital for study follow-up.

**Theoretical Framework**

The theoretical framework for this investigation was based on the revised symptom management conceptual model (Dodd et al., 2001). The conceptual model has three dimensions: symptom experience, symptom management strategies, and outcomes, which are influenced by the recognized domains of nursing science, person, health/illness, and environment. The individual’s response to a symptom includes physiologic, psychologic, sociocultural, and behavioral components, and one or more of any of these responses may be seen in a single symptom. Patients with cancer and HF experience multiple symptoms, and outcomes are determined by the management strategies implemented in response to the symptom experience reported by the patient.

## INSTRUMENT AND MEASURES

**The MDASI-HF Symptom Instrument**

The MDASI-HF (Figure 1) is a 27-item self-report assessment instrument developed specifically for patients with cancer and concurrent HF (Fadol et al., 2008). The MDASI-HF instrument has been tested with a reliability of α = .89 (13 symptoms), α = .83 (8 HF-specific items), and α = .92 (interference items). Criterion-related validity with the Eastern Cooperative Oncology Group performance scale (*r* = 0.63) and the New York Heart Association classification (*r* = 0.65) were statistically significant (*p* = .01; Fadol et al., 2008). In the validation of the MDASI-HF instrument, the reliability of the 13 core symptoms, 8 HF-specific items, and the 6 interference items were .91, .76, and .86, respectively.

**Figure 1:**
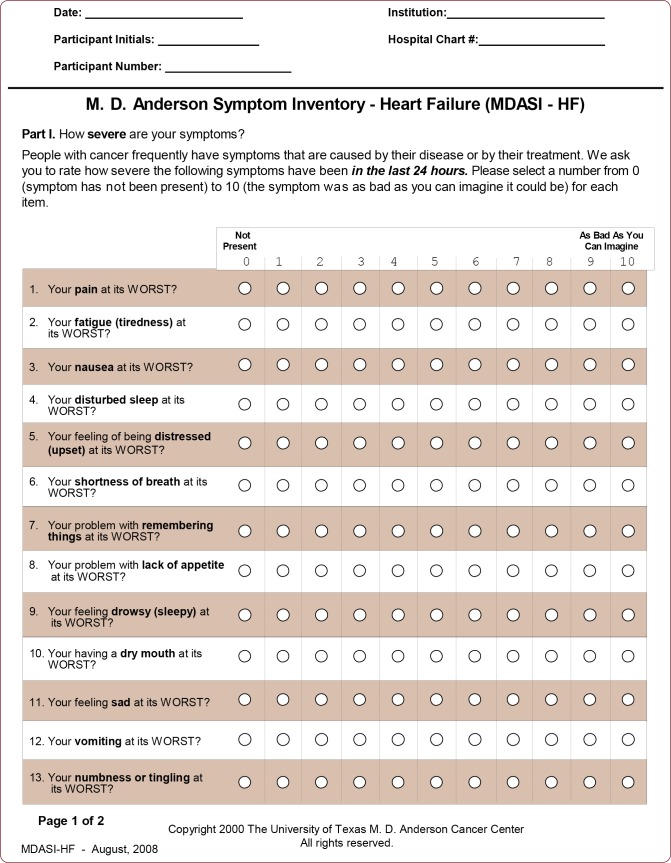
The MD Anderson Symptom Inventory–Heart Failure Instrument

**Figure 1:**
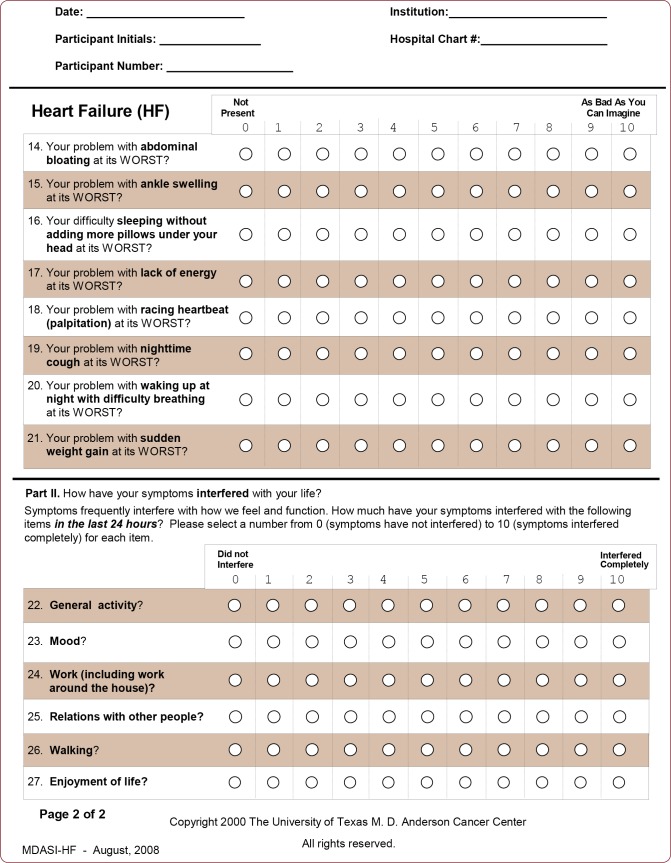
The MD Anderson Symptom Inventory–Heart Failure Instrument

Each symptom in the MDASI-HF was rated on an 11-point scale (0–10) to indicate the presence and severity of a symptom, with 0 meaning "not present" and 10 meaning "as bad as you can imagine" in the last 24 hours. The MDASI-HF also includes ratings on how symptoms interfered with the different aspects of a patient’s life in the last 24 hours. The interference items were also measured on a 0 to 10 scale with 0 being "did not interfere" to 10 "interfered completely." The mean of all these symptom interference items was used as a measure of overall symptom distress.

As agreed by the cardiologists and the oncologists in the group, the threshold for the MDASI-HF symptom scores that generated an alert were as follows: pain = 5; shortness of breath = 5; fatigue = 7; nausea = 7; disturbed sleep = 7; distressed or upset = 7; problem with remembering = 7; lack of appetite = 7; drowsy = 7; dry mouth = 7; feeling sad = 7; vomiting = 7; numbness or tingling = 7; and heart failure–specific symptoms = 7.

## PROCEDURES

**Figure 2:**
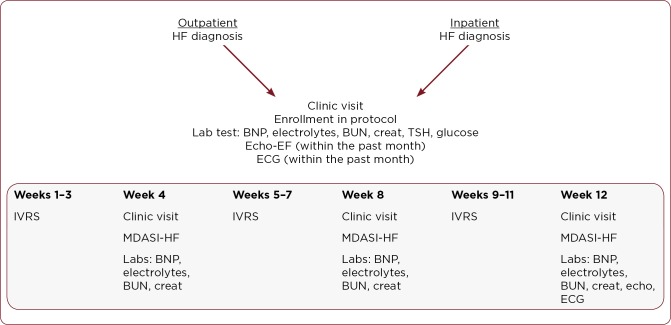
MD Anderson Symptom Inventory–Heart Failure symptom management protocol. HF = heart failure; BNP = B-type natriuretic peptide; BUN = blood urea nitrogen; creat = creatinine; ECG = electrocardiogram; echo = echocardiogram; EF = ejection fraction; IVRS = interactive voice response system; MDASI-HF = MD Anderson Symptom Inventory–Heart Failure.

Participants were recruited from the inpatient units and the outpatient heart failure clinic. Following baseline assessment and screening, participants who met inclusion criteria met with the principal investigator and a trained research nurse who provided detailed information about the study and answered questions before the participants signed the informed consent form. The participants were then enrolled in the protocol as shown in Figure 2. The research nurse explained to the participants how to complete the MDASI-HF instrument using pencil and paper based on a self-assessment of their symptoms for the past 24 hours. Thereafter, the research nurse provided detailed instructions regarding the IVRS, followed by a return demonstration on using the IVRS to ensure understanding. Participants were informed that they will receive a preprogrammed telephone call once a week at the day and time of their choosing at the telephone number they had provided.

**The Interactive Voice Response System**

**Figure 3:**
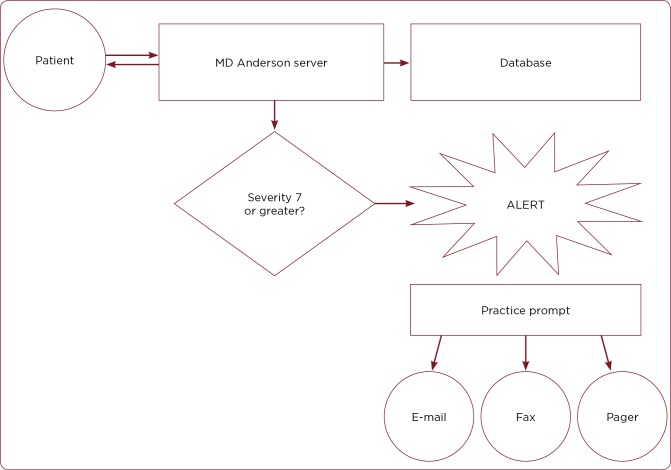
Schema of the MD Anderson Interactive Voice Response System

While patients were at home, symptom(s) were monitored using the MDASI-HF questionnaire preprogrammed via the IVRS as shown in the schematic diagram (Figure 3). The IVRS was delivered to the patient with an automated voice asking the same questions as on the MDASI-HF questionnaire. If there was no answer to the first call, the automated system generated two more calls at 30-minute intervals. If there was no answer on the scheduled day chosen by the participant, or the call was answered but the participant was unavailable, the system called again the next day repeating the same cycle. After 2 consecutive days of no response after the scheduled day, the research nurse contacted the participant by telephone.

**Figure 4:**
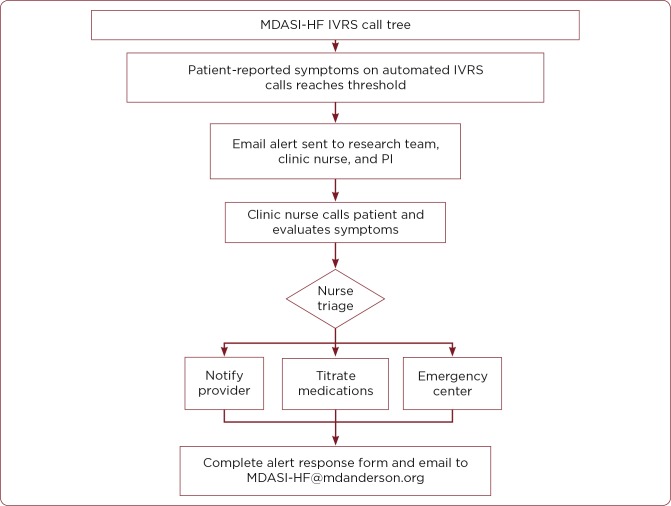
MD Anderson Symptom Inventory–Heart Failure interactive voice response system call tree. IVRS = interactive voice response system; MDASI-HF = MD Anderson Symptom Inventory–Heart Failure; PI = principal investigator.

The automated telephone calls were generated to call the participant’s home phone number on a weekly basis for 3 months while enrolled in the study. When a participant’s reported symptom(s) reached critical threshold levels, the program automatically generated an alert that prompted the clinic nurse. During office hours (Monday through Friday; 8 am to 5 pm), the symptom alerts were sent via electronic mail to the IVRS nurse who then triaged the patient’s response per protocol, and consulted with the physician when necessary and initiated interventions as appropriate (Figure 4). If the symptom alert occurred after office hours or on weekends, the alerts were forwarded via electronic mail through the pager of the cardiologist on call. All participants were instructed to access the IVRS anytime if symptoms became worse.

**Data Entry and Analyses**

The demographic characteristics and MDASI-HF symptom assessment scores completed by the participants using pencil and paper during clinic visits were manually scanned and uploaded in the database. The participants’ responses using the IVRS were downloaded and imported to the IBM Statistical Package for the Social Sciences (SPSS) Statistics version 227.0.

Descriptive statistics were used to describe the ratings of symptom severity and symptom interference with daily function. Demographic and disease characteristics were summarized using means, standard deviations, ranges, count, and proportions. Paired t-tests were used to examine symptom reduction from baseline to the end of 3 months.

## RESULTS

A total of 26 participants who met the inclusion criteria agreed to participate in the study. The demographic characteristics are listed in Table 1. A majority of the participants (80.8%) were ≤ 65 years with a mean age of 57.6 years (± 11.5 years), female (61.5%), and had some college or graduate education (54%).

**Table 1:**
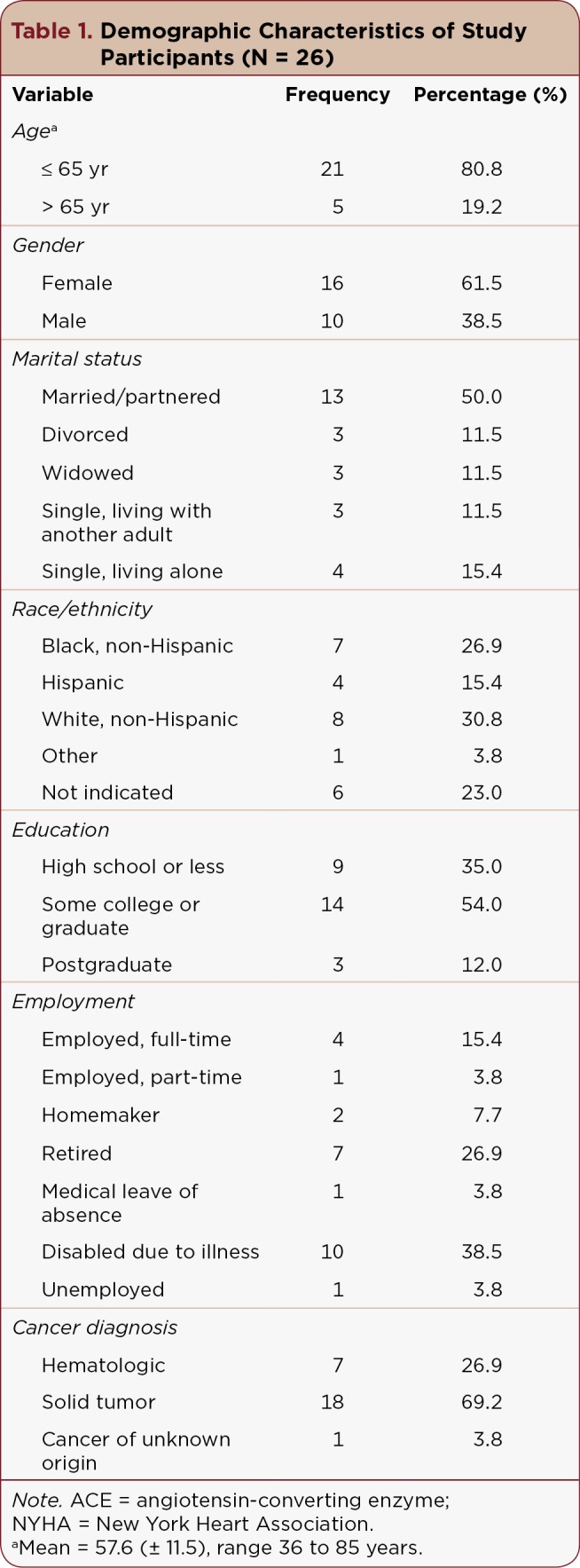
Demographic Characteristics of Study Participants (N = 26)

**Table 1:**
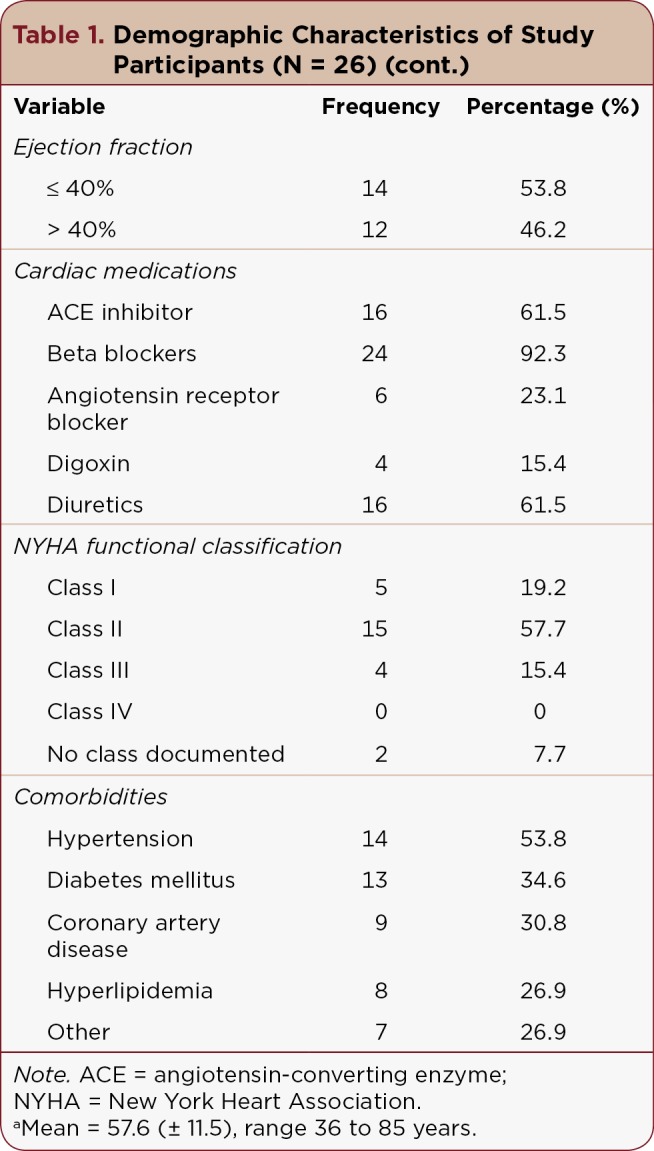
Demographic Characteristics of Study Participants (N = 26) (cont.)

Most of the participants (53.8%) had HF with systolic dysfunction (left ventricular ejection fraction ≤ 40%) and were on recommended HF medications of beta blockers (92.3%), angiotensin-converting enzyme inhibitors (61.5%), angiotensin receptor blockers (23.1%), and diuretics (61.5%). The participants had multiple comorbidities, including hypertension (53.8%), diabetes mellitus (34.6%), coronary artery disease (30.8%), hyperlipidemia (26.9%), and other comorbid conditions (26.9%).

Fourteen (54%) participants completed the 3-month follow-up for the study with average IVRS usage rates of 84% at 1 month and 82% at 3 months. Twelve participants were not able to complete the 3-month study follow-up for multiple reasons (3 were hospitalized for fever, acute renal failure, infection, and sepsis secondary to cancer treatment; 4 were transitioned to hospice; 1 had brain surgery and requested to discontinue participation in the study; 1 transferred care to another institution after a coronary artery bypass surgery; and 3 participants were lost to follow-up, and could not be reached after not responding to IVRS calls).

**Symptom Alerts**

Over the course of the IVRS monitoring, 152 IVRS calls were completed and 107 critical threshold alerts were generated, prompting triage and physician notification, medication titration, and unscheduled clinic visits. Most of these alerts were managed by telephone, particularly those related to diuretic titration. Nine (34%) of the participants received intervention for their reported symptoms in the outpatient clinic or in the emergency center. The interventions included antibiotics (30.8%), diuretics (19.2%), blood transfusion (11.5%), and opioids (7.7%).

The symptoms that generated the most alerts were pain (12.3%), fatigue (5.8%), shortness of breath (5.8%), and lack of energy (5.8%) as shown in Table 2. Some participants generated more than one threshold alert.

**Table 2:**
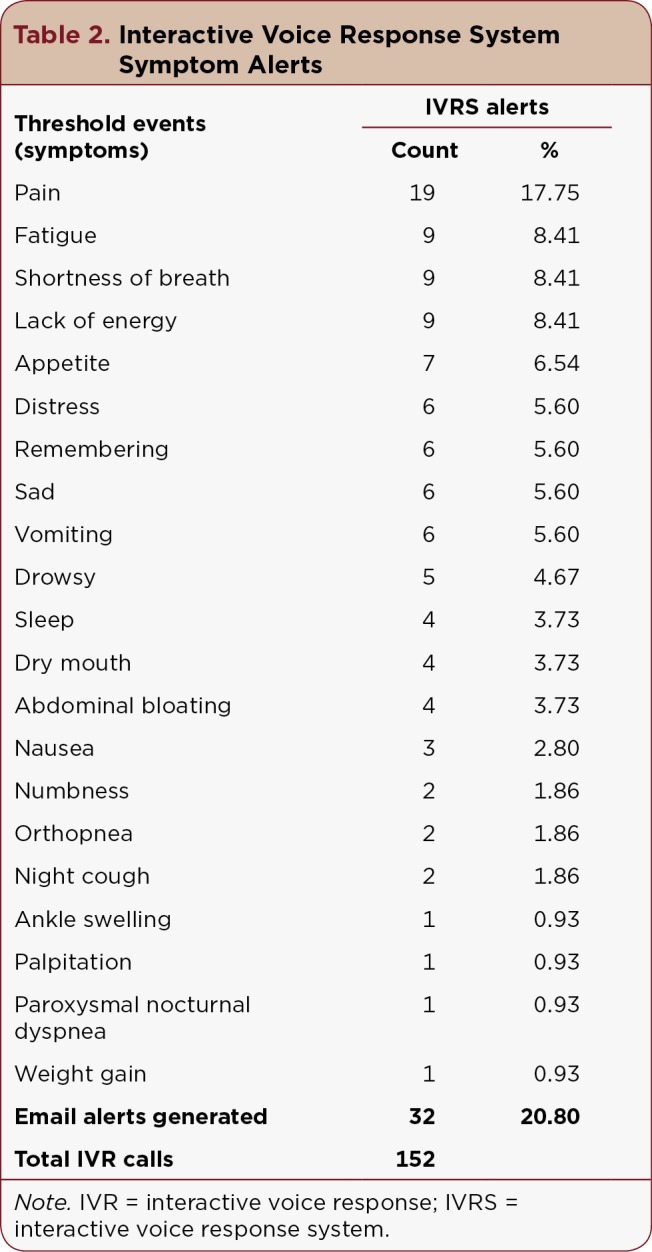
Interactive Voice Response System Symptom Alerts

**Symptom Scores**

With paired t-tests, no statistically significant difference was observed in the symptom scores of the 14 patients who completed the study at the end of 3 months compared to baseline (*p* < .002) after Bonferroni adjustment (Table 3). This included the mean symptom scores for all heart failure symptoms: both overt symptoms (nighttime cough, waking up at night with difficulty breathing, lack of energy, difficulty sleeping without adding pillows [orthopnea], racing heart beat) and covert symptoms (sudden weight gain, abdominal bloating).

**Table 3:**
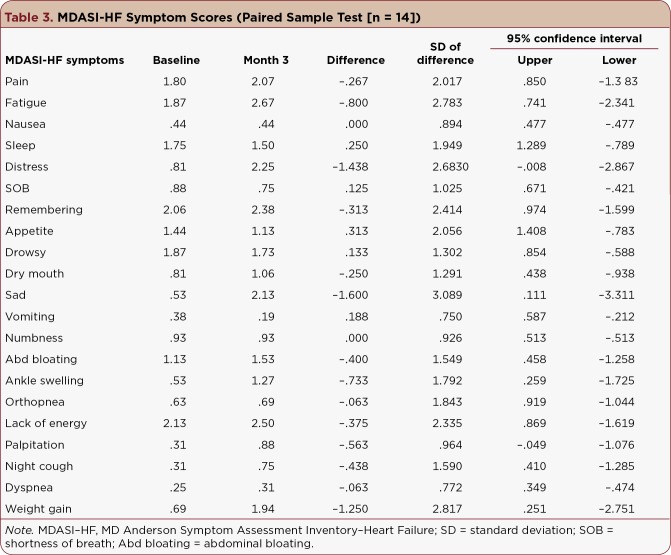
MDASI-HF Symptom Scores (Paired Sample Test [n = 14])

## DISCUSSION

Participants who had low complication rates used the IVRS for symptom monitoring over the 3-month duration of the study. This resulted in timely intervention to prevent worsening of symptoms. Unfortunately, about half of the sample population developed severe complications due to worsening of the cancer, requiring hospitalization or resulting in admission to hospice and were unable to complete the study. Cancer patients who developed HF while receiving cancer treatment had additional comorbid conditions that resulted in further clinical deterioration, requiring hospital admission for clinical management. The symptoms reported by patients that required immediate intervention are clinical manifestations of the overlapping of cancer, HF, and other comorbidities. This is evidenced by the interventions (antibiotics, diuretics, blood transfusions, and opioids) patients received upon presentation in the emergency center or in the clinic.

The characteristics of the study sample are similar to those of the general HF population (Roger et al., 2011), except for age. Participants in this study were younger (mean age = 57.6 years) compared to the general population with HF (mean age = 65 years). The majority of the participants (69%) in this study had solid tumors (i.e., breast, prostate, colorectal, gastric, and lung cancer) diagnosed at a younger age and subsequently developed HF as a result of chemotherapy-induced cardiomyopathy.

The majority of patients in this study had a satisfactory performance status classified as New York Heart Association (NYHA) Class II at study enrollment with low mean symptom scores. This was related to the fact that most of the participants were enrolled in the study just prior to hospital discharge after they were placed on optimum HF pharmacologic therapy and were clinically euvolemic. Moreover, the participants were closely monitored with the IVRS while enrolled in the study. A randomized controlled trial comparing IVRS with usual care is needed to provide information regarding the usefulness of IVRS monitoring in patients with a dual diagnosis of cancer and HF.

To date, there are no published studies using a validated symptom assessment instrument via the IVRS for symptom monitoring of cancer patients with HF. Prior HF trials (Chaudhry et al., 2010; Inglis et al., 2010, 2011; Kashem, Cross, Santamore, & Bove, 2006) that have evaluated structured telephone support for patients did not use a symptom assessment instrument for monitoring of symptoms, and patients with concurrent cancer diagnoses were not included in the study.

Telemonitoring using the IVRS can be a useful tool for the remote surveillance of patients with cancer and HF. With timely notification, clinicians can intervene early given the evidence of clinical deterioration. Although prior HF studies on telemonitoring showed contradicting results in the study endpoints (hospital readmission, mortality; Chaudhry et al., 2010; Desai, 2012; Inglis et al., 2010) it must be taken into consideration that the efficacy of complex interventions such as telemonitoring depends on the context in which they are applied (Clark, Savard, & Thompson, 2009; Craig, 2008). Patients with cancer who developed subsequent HF with overlapping symptoms resulting from both conditions have not been investigated, but may benefit from telemonitoring.

Symptoms reported by patients with cancer and HF require an in-depth investigation of the etiology to formulate a list of differential diagnosis to guide decision-making and intervention. With the advances in cancer treatment and expansion of cancer therapies to more elderly individuals with a greater burden of comorbidities (Aapro et al., 2011; Serrano et al., 2012; Tarantini et al., 2012), there is a greater need for providers to enlarge their focus in the management of cancer patients to include pre-existing chronic illnesses as well as cancer treatment–related illness and disability.

For example, when an HF patient without cancer reports increased shortness of breath and worsening of lower extremity edema, these symptoms are usually secondary to volume overload. However, in a patient with cancer and HF, the symptom has to be investigated for other etiologies in addition to volume overload. Shortness of breath may indicate lung metastasis or pulmonary embolism, particularly in patients actively receiving cancer treatment such as bevacizumab (Avastin). Lower extremity edema in a cancer patient is more than just volume overload. Differential diagnosis should include evaluation for lymphedema, pelvic tumors, deep venous thrombosis, and hypoalbuminemia in addition to volume overload.

Symptom monitoring with telemonitoring services can increase patients’ access to health-care providers, crossing geographic barriers and provide a surveillance mechanism for the early detection of patients who are getting into difficulties, and focusing scarce resources on patients with the greatest need for intervention. Given the aging of the population and an increasing number of new anticancer treatments with potential cardiotoxicity, the number of patients who will develop HF will potentially increase in the years to come. Thus, innovative strategies to improve the management of these patients need to be evaluated to improve the care and quality of life of these patients, as well as the economic benefit to the health-care system given limited resources.

## DIRECTIONS FOR FUTURE RESEARCH

Future studies on symptom monitoring using IVRS should consider starting enrollment of participants at the emergency center admission to evaluate its impact on hospital readmission, which was not measured in this pilot study. Additional research is needed to determine if symptom monitoring using the IVRS is comparable to other methods of symptom monitoring such as using the internet or mobile devices. A randomized controlled trial to compare telemonitoring with other forms of mobile technology should be conducted in patients with cancer and HF. Study endpoints should address medical costs, 30-day hospital readmission, mortality, health-related quality of life, and patient satisfaction. Given that the Centers for Medicare and Medicaid Services is under considerable pressure to restrain costs for the delivery of health-care services to beneficiaries covered by its program, innovative strategies such a telemonitoring should be explored in the long-term management of these patients. Without a clear understanding of the economic implications of telemonitoring interventions in this specific patient population, it will be difficult to inform payers on the reimbursement of this program. Although the IOM’s report "Crossing the Quality Chasm: A New Health System for the 21st Century" (2001) states that information technology must play a role in the redesign of the health-care system to improve the quality of care for patients with chronic diseases such as cancer and HF, a comparative study with other forms of technology for delivering care to these patients should be explored.

Moreover, future IVRS trials should include Spanish-speaking patients, given that these patients are a rapidly growing group in the United States population, with a high incidence of cancer and coronary artery disease, a significant risk factor for HF.

## IMPLICATIONS FOR PRACTICE

The use of a system like the MDASI-HF IVRS for symptom monitoring in patients with cancer and HF has the potential to (1) assist providers with the early identification of HF symptoms related to the cardiotoxic effects of cancer therapy and to evaluate the effectiveness of treatment interventions; (2) allow for identification of specific symptom severities, which can be immediately flagged and monitored with a telephone call or a more frequent follow-up visit to prevent further exacerbation of symptoms; (3) assist providers in following the clinical status of patients over time and incorporating symptoms severity in making treatment decisions; and (4) improve quality of care through prevention of unnecessary hospitalization, thereby improving patient satisfaction and quality of life.

## CONCLUSIONS

Although this pilot study did not show statistically significant improvement in symptom scores, it has demonstrated the interest of patients for a longitudinal symptom monitoring. This study provides preliminary data for symptom monitoring in patients with cancer and HF, leaving questions about the effectiveness of telemonitoring in this specific patient population. Given that cancer patients with HF usually have poor functional status and an increased number of symptoms over time, and are often unable to drive themselves to the clinic or hospital, telemonitoring may facilitate remote symptom management. Further research is needed to elucidate approaches that will have the most economic impact as well as greatest improvement of the quality of life of these patients.

**Acknowledgment**

The authors acknowledge the contributions of research nurses Mona Massey and Cindy Chua for data collection, and Dr. Charles Cleeland, Chair of Department of Symptoms Research, for his insight and critique in the development of this study.
